# Machine Learning–Based Predictive Model for Functional Independence in Spinal Cord Injury: Protocol for a Predictive Rule Development and Validation Study

**DOI:** 10.2196/95236

**Published:** 2026-07-03

**Authors:** Alberto Isaac Perez-Sanpablo, Marlene Alejandra Rodriguez-Barragan, Alicia Meneses-Peñaloza, Jimena Quinzaños-Fresnedo, Aida Barrera-Ortiz, Fabiola Monserrat Palomino-Ramos, Oscar Prado-Escobar, Marcela D Rodríguez, Irvin Hussein Lopez-Nava, Jose Ambrosio-Bastian

**Affiliations:** 1 Motion Analysis and Rehabilitation Engineering Lab, Instituto Nacional de Rehabilitación, , Mexico City, Mexico; 2 Neurologic Rehabilitation Division, Instituto Nacional de Rehabilitación, Mexico-Xochimilco Av. 289, Mexico City, 14389, Mexico, 52 5559991000 ext 13410; 3 Pediatric Rehabilitation Division, Instituto Nacional de Rehabilitación, Mexico City, Mexico; 4 Faculty of Engineering, Universidad Autónoma de Baja California, Mexicali, Baja California, Mexico; 5 Data Science and Machine Learning Lab, Centro de Investigación Científica y de Educación Superior de Ensenada (CICESE), Ensenada, Baja California, Mexico; 6 Universidad Tecnológica de México (UNITEC), Mexico City, Mexico

**Keywords:** spinal cord injury, prognosis, machine learning, functional independence, SCIM-III, predictive modeling, rehabilitation, artificial intelligence, trunk control

## Abstract

**Background:**

Spinal cord injury (SCI) causes substantial disability by disrupting spinal pathways, making functional independence a central rehabilitation goal. In 2024, approximately 15.4 million people worldwide were living with SCI. Despite its clinical relevance, traditional prognostic tools, notably the International Standards for Neurological Classification of Spinal Cord Injury (ISNCSCI), have limitations. Furthermore, access to advanced diagnostics constrains prediction. Existing models often fail to estimate independence, limiting goal setting and resource planning.

**Objective:**

This study aims to develop and validate machine learning (ML)–based rules that predict functional independence, as measured by the Spinal Cord Independence Measure, version III (SCIM-III), at 3, 6, and 12 months post injury in individuals with SCI. The model combines clinical admission predictors, readily available to enhance predictive performance: age, sex, time since injury, upper and lower extremity strength, ISNCSCI data (American Spinal Injury Association Impairment Scale grade and neurologic level of injury), rehabilitation type, and the Trunk Control Scale score validated by the research team.

**Methods:**

Using retrospective electronic clinical records (2015‐2026) from Mexico’s Nacional de Rehabilitación Luis Guillermo Ibarra Ibarra (INR LGII; a tertiary national rehabilitation reference center) for model development and a prospective independent cohort (October 2026-October 2027) for external validation, the study will develop ML models to accurately and generally predict SCIM-III outcomes. Eligible participants are adults (≥18 years of age) with subacute or chronic SCI. Six architectures are compared: linear regression, classification and regression tree, categorical boosting (gradient boosting algorithm), light gradient boosting machine, multilayer perceptron, and Gaussian process regression, using 10-fold stratified cross-validation for internal validation. Performance is assessed by root-mean-square error, mean absolute error, *R*², area under the curve, calibration plots, and decision curve analysis. The approach prioritizes reproducibility, interpretability, and clinical applicability, addressing prior models’ limitations (small sample sizes, limited validation, and impractical input requirements). Key steps include data preprocessing, feature selection, model training with appropriate algorithms, and robust evaluation against existing prognostic benchmarks. External validation and ethical considerations are integrated, with commitments to data sharing where feasible.

**Results:**

The study was approved and funded in October 2025. The retrospective development cohort targets 500 registries (from 2025‐2026) collected between October 2025 and June 2026. A total of 119 records were processed by March 2026. ML development is planned between June 2026 and March 2027. The external prospective validation cohort (n=100) is planned for October 2026-October 2027, and validation for June 2027-March 2028. Results are expected by January 2028.

**Conclusions:**

A reliable, clinically actionable ML-based prediction tool that estimates SCIM-III trajectories to support goal setting, rehabilitation planning, and resource allocation in SCI care will be developed. Model performance will be benchmarked against published SCI prediction rules, and it will be considered clinically useful if it outperforms the mean-score baseline and existing rules on the prospective validation cohort.

## Introduction

### Overview

Spinal cord injury (SCI) is an important cause of disability due to the impairment of the ascending and descending pathways of the spinal cord. This is why effective SCI rehabilitation includes patient-centered goal setting by the patient, their family, and the multidisciplinary team attending to them. Goal setting helps identify a person’s needs, values, and expectations and is ultimately regarded as a central principle in rehabilitation. Nevertheless, there is some controversy about how to establish goal setting [1]. Prognostic tools can objectively help establish those goals, as previously demonstrated [2].

In 2026, the principal clinical tool for predicting recovery and function is the International Standards for Neurological Classification of Spinal Cord Injury (ISNCSCI). Nevertheless, those standards have some limitations in their prediction properties [3]. In particular, a ceiling effect for mild injuries (American Spinal Injury Association Impairment Scale [AIS] D), and the potential for misclassification at the time of the initial examination or follow-up have been described. Other studied prediction works use magnetic resonance imaging (MRI), cerebrospinal fluid biomarkers, and neurophysiological measures, which are difficult to access for many care centers and are typically performed in the first hours following the injury, a situation that does not usually occur in many places [4].

Clinical prediction rules, a subset of clinical indicators to predict outcomes or assist in clinical decisions, have been developed as prognostic tools [5]. The majority of them focus on independent walking prediction, and use regression models that are not the best prediction model [6,7]. A recent study focuses on independence and uses a classification regression tree. Still, the main outcome is the Functional Independence Measure score, which is not specific to people with SCI, and whose study population was limited to individuals with cervical SCI, which undermines its applicability [8].

Machine learning (ML) helps find a predictive model that better fits reality [9]. Also, using specific, validated tools is necessary to make a prognosis after an SCI. So, the main objective of our work is to develop and validate ML-based rules predicting Spinal Cord Independence Measure, version III (SCIM-III) at 3, 6, and 12 months in individuals with SCI.

### Background

Neurological diseases are the leading cause of disability worldwide, and in Mexico, SCI is among the most frequent conditions requiring rehabilitation care. SCI is one of the most complex pathologies in rehabilitation medicine due to its direct impact on functionality, independence, and quality of life of affected individuals [10]. People with this diagnosis, many of whom are working age, would benefit from precise prognoses to guide rehabilitation goals, optimize resources, and improve patient-caregiver communication. However, health inequalities may affect SCI rehabilitation outcomes in Mexico: lower-income and rural populations may face delayed access to specialized centers such as Instituto Nacional de Rehabilitación Luis Guillermo Ibarra Ibarra (INR LGII). These disparities may influence functional trajectories and are considered in the interpretation of model generalizability.

Although these variables have been used as inputs in functional prediction models under development in 2026, clinical decision-making still depends largely on individual clinical judgment, which may lead to uncertainty in goal setting, institutional burden, or uncertainty for patients and caregivers [11]. Some clinical rules allow partial anticipation of functional recovery, in particular concerning walking ability, but few have demonstrated a significant effect in clinical practice [6,7,12,13]. Several studies have identified that variables such as injury severity classification (AIS), neurological level of injury, Upper Extremity Motor Score (UEMS), Lower Extremity Motor Score (LEMS), and Trunk Control Scale (ECT) have prognostic value [6,7,12,13]. However, there are few tools capable of predicting overall functional independence, as comprehensively measured by the SCIM-III [14].

In the multidisciplinary management of individuals following SCI, it is essential to have tangible, measurable, and objective tools to estimate functional prognosis in the short, medium, and long term. Several approaches currently allow preliminary predictions based primarily on the severity and neurological level of injury, using the American Spinal Injury Association classification or the AIS. However, despite their clinical usefulness, these measures alone may not fully capture the complexity of functional recovery, highlighting the need for objective and reproducible prognostic tools that can support clinical decision-making and rehabilitation planning.

MRI plays a fundamental role in the evaluation of SCI, as it allows detailed assessment of spinal cord involvement and surrounding soft tissues, enabling the identification of the severity of neurological damage and the potential for recovery. In this context, MRI-based scoring systems, such as the Brain and Spinal Injury Center score, have demonstrated utility in predicting neurological outcomes in individuals with acute SCI.

Other predictive strategies include the timing of posttraumatic surgical intervention, as multiple studies and meta-analyses have demonstrated that early surgical decompression (within the first 24 hours) is associated with a higher probability of neurological improvement as measured by the AIS.

In addition, several structural and neuroinflammatory biomarkers related to SCI have been identified. These biomarkers are derived from disruption of the blood-spinal cord barrier and from inflammatory processes occurring after the injury. Among the most studied are neurofilaments, glial fibrillary acidic protein, phosphorylated neurofilament heavy chain, and other proteins detected in cerebrospinal fluid and blood, whose concentrations have been correlated with injury severity and the potential for neurological recovery. These findings suggest that biomarkers may contribute in the future to improving the prediction of functional outcomes in individuals with SCI.

Although neurological assessment using ISNCSCI and the AIS classification allows estimation of SCI severity and provides guidance regarding neurological prognosis, their ability to predict overall functional independence remains limited. Tools such as the SCIM-III were developed specifically for SCI to provide a more comprehensive evaluation of functional performance in activities of daily living. However, although these instruments allow the assessment of functional status in individuals with SCI, their capacity to accurately predict future functional independence remains limited, which has motivated the development of more advanced predictive models capable of integrating multiple clinical and functional variables into objective prognostic frameworks [15,16].

ML has been applied in the field of medicine for various purposes, including disease diagnosis, drug development, and the analysis of medical data. This approach offers several advantages in the prediction and development of prognostic models for certain diseases compared with traditional statistical methods, such as linear or logistic regression. Specifically, it imposes fewer restrictions on the number of predictors derived from a given dataset and is well-suited for identifying complex nonlinear relationships within data.

In the context of SCI, ML has already been applied in studies focusing on quality of life, duration of opioid prescription, length of stay in intensive care units, and, more recently, in the prediction of walking outcomes [17].

This new wave of research studies the highly complex problem of predicting personalized rehabilitation trajectories, using multimodal, longitudinal, and often incomplete datasets. Such an approach has high potential clinical and social impact and represents a transition toward computational evidence-based individualized medicine.

Despite the growing data supporting these approaches, no studies conducted in Mexico or Latin America have validated a predictive rule for the SCIM-III score or integrated the ECT developed by members of our research group [13]. At the INR LGII, trunk control assessment is a key component of treatment planning. Trunk control has been identified as an accurate predictor of functional independence, demonstrating high sensitivity and specificity [13].

The clinical ECT developed at our institution has led to subsequent research conducted at our center, which demonstrated its prognostic validity for predicting functional independence and walking ability in individuals with SCI, highlighting trunk stability as a relevant clinical indicator of functional outcomes. These findings support the integration of trunk control assessment into prognostic models of rehabilitation outcomes.

In addition, recent technological advances have expanded the possibilities for a more objective evaluation of trunk function. The validation of inertial measurement units for the assessment of trunk control in individuals with SCI has shown that wearable sensors can provide reliable and quantitative measurements of postural control during functional tasks.

Together, these advances reinforce the potential for integrating traditional clinical assessments with technological tools to improve prognostic accuracy. Based on these institutional achievements, our center is well-positioned to contribute to the development of clinical prediction rules for functional independence in SCI, integrating traditional neurological variables with trunk control assessment and emerging quantitative tools [13,18].

### Hypothesis and Research Question

Previous research has identified several early clinical variables associated with neurological and functional recovery following SCI. Factors such as age, motor strength in key myotomes, sensory preservation in specific dermatomes, and the presence of sacral sparing (S4-S5) have consistently been reported as relevant indicators of neurological recovery and long-term functional outcomes. In particular, certain myotomes and dermatomes have special clinical relevance for predicting walking ability. Among the most important myotomes are L3 (quadriceps femoris, responsible for knee extension) and S1 (gastrocnemius-soleus complex, responsible for plantar flexion), as muscle strength in these groups is closely associated with the ability to support body weight and generate the propulsion required for ambulation. From a sensory perspective, the L3 and S1 dermatomes, assessed through light touch and pinprick sensation, have also demonstrated prognostic value in several clinical models of walking recovery. Likewise, the preservation of sensation in the sacral segments S4-S5, known as sacral sparing, represents one of the most important predictors of neurological improvement, as it reflects the integrity of residual neural pathways below the level of injury [6,10,11,19].

However, our research group has demonstrated that global independence is more relevant than walking ability in determining quality of life [13]. The ECT developed at the INR LGII has shown high correlations with functional outcomes and may improve predictive performance, since the ISNCSCI does not evaluate trunk musculature [10,13]. Therefore, combining this scale with artificial intelligence techniques could lead to the development of a new, more accurate, and clinically useful prediction rule.

This leads to the following hypothesis: it is possible to develop a reliable and clinically relevant functional prediction rule to estimate the SCIM-III score in individuals with SCI using clinical variables such as age, time since injury, upper and lower extremity strength (UEMS and LEMS), and trunk control through the use of multivariate statistical models and ML algorithms. This hypothesis gives rise to the following research question: Is it feasible to develop a consistent and valid functional prediction rule to estimate the SCIM-III score in individuals with SCI based on selected clinical variables and using ML models?

### Justification

At the INR LGII, approximately 2500 participants from across the country are treated annually, primarily individuals of working age who may benefit from a more accurate prognosis to plan rehabilitation goals, optimize resources, and improve communication of expectations [20]. Combining artificial intelligence with validated clinical variables in a predictive model will not only fill an important knowledge gap in Mexico but will also help advance neurological rehabilitation, strengthen health equity, and promote the training of specialized human resources. This project could benefit this population, as well as 30 specialist physicians and trainees, institutions, and professionals across the country involved in SCI rehabilitation, and more than 20,000 professionals in Mexico working in neurological rehabilitation.

The proposal is consistent with the objectives of the Basic and Frontier Science 2025 call by generating original frontier knowledge in a critical and rapidly growing health field, using ML to predict functional outcomes in SCI participants.

### Objectives

The main objective of this work is to develop and validate ML-based rules predicting independence, as measured by SCIM-III, at 3, 6, and 12 months post injury in individuals with SCI. Secondary objectives include:

- To build a database of clinical variables in clinical records of individuals with SCI readily available in low-resource rehabilitation settings, such as age, time since injury, upper and lower extremity strength (UEMS/LEMS), trunk control, and ISNCSCI data
- To identify the best predictors using statistics and ML models.
- To select and validate the best-performing model, generating a practical clinical prediction rule for functional independence estimation.
- To disseminate results through open-access publications, conference presentations, and scientific training activities for rehabilitation students.

## Methods

### Study Design

This is an observational, analytical, mixed design (retrospective-prospective) study. The study consists of two components: (1) a retrospective review of existing electronic clinical records of individuals with SCI treated at INR LGII, and (2) a prospective follow-up with a 12-month SCIM-III assessment.

### Setting

The study will be conducted at the INR LGII, a tertiary care national reference center for rehabilitation medicine in Mexico City, Mexico. The INR LGII receives individuals from all regions of the country and is the largest SCI rehabilitation center in Mexico.

### Participants

Textbox 1 presents the inclusion and exclusion criteria of participants throughout the study phases.

Textbox 1. **Inclusion and exclusion criteria for all study phases.**

**Inclusion criteria for the retrospective phase**

- Age ≥18 years at time of spinal cord injury (SCI)
- Confirmed diagnosis of subacute or chronic SCI (traumatic or nontraumatic)
- Clinical follow-up at the Instituto Nacional de Rehabilitación Luis Guillermo Ibarra IbarraI with evaluations at 3, 6, and/or 12 months post admission to rehabilitation
- Complete clinical records with the following variables: Spinal Cord Independence Measure, version III, American Spinal Injury Association Impairment Scale grade, Upper Extremity Motor Score, Lower Extremity Motor Score, Trunk Control Scale, age, sex, type of rehabilitation, and time since injury

**Inclusion Criteria for the Prospective Phase**

- Age ≥18 years at time of SCI
- Confirmed diagnosis of subacute or chronic SCI (traumatic or nontraumatic)
- Signed informed consent

**Exclusion criteria for retrospective and prospective phases**

- Confirmed diagnosis of severe comorbid neurological conditions independent of SCI (eg, stroke, traumatic brain injury, and neurodegenerative disease)
- No motor deficit attributable to SCI
- Age <18 years

### Elimination Criteria for Both Phases

The following elimination criteria will apply to both retrospective and prospective phases:

- Records with more than 30% missing data for the defined predictor variables.
- Records with serious data entry inaccuracies or errors preventing reliable analysis.
- Records duplicated.
- Withdrawal of consent during the prospective data collection stage.

### Sample Size

Our target is a total of at least 500 patient records for internal development and validation, plus a separate external temporal validation cohort of 100 new patients (prospectively recruited from October 2026 to October 2027). Unlike hypothesis-testing sample size calculations, ML validation studies aim to estimate predictive performance accurately (low bias) and precisely (narrow CIs). Most studies on clinical ML-based prediction models do not justify the sample size [21], since there is no universally accepted standard method for sample size in ML. To address this gap, we adopted a pragmatic, mixed methods approach to justify the sample size based on:

- Model complexity and number of predictors*:* our nonlinear algorithms (gradient boosting and neural networks) require larger sample sizes than regression models [22,23]. With up to 9 predictors, a sample of 500 patients provides a predictor-to-sample ratio of >50:1. This ratio exceeds those generally proposed for ML studies, as well as those used in clinical prediction studies (eg, 20:1 or 37:1 from [24]). Consequently, this ratio is sufficient to learn meaningful patterns without memorizing noise, thus reducing the risk of overfitting.
- Validation needs: based on common practices in clinical ML studies, recent research has used 15%‐30% of the data for external validation [25,26]. Accordingly, we defined 20% (100 patients) as the external test set. This proportion allows us to evaluate the final model performance on unseen data without compromising the training or hyperparameter validation phases.

### Variables

#### Outcome Variable

The primary outcome variable will be the SCIM-III score at 3, 6, and 12 months post admission. SCIM-III is a validated SCI-specific measure of functional independence, with higher scores reflecting greater independence [16].

#### Predictor Variables

Predictor variables, definitions, and measurement scales used for model development are shown in Table 1.

**Table 1.** Candidate predictor variables, clinical definitions, and encoding strategies for model development.

Variable	Description	Scale/units
Age	Age at SCI^a^ onset	Years (continuous)
Sex	Biological sex	Male/female (binary)
Time since injury	Months from SCI to rehabilitation admission	Days (continuous); normalized (*z* score)
AIS^b^ grade	American Spinal Injury Association Impairment Scale (neurological completeness)	A, B, C, D; one-hot encoded for model training
UEMS^c^	Upper Extremity Motor Score (ISNCSCI^d^)	0‐50 (continuous)
LEMS^e^	Lower Extremity Motor Score (ISNCSCI)	0‐50 (continuous)
ECT^f^	Trunk Control Scale (developed at INR LGII)^g^	0‐23 (ordinal, treated as continuous numeric)
Rehabilitation type	Type of rehabilitation program received	Categorical
Neurological level of injury	Cervical, thoracic, lumbar, or sacral level as determined by ISNCSCI examination at admission	Categorical (ordinal descending C1→S5); encoded as ordinal integer

a SCI: spinal cord injury.

b AIS: American Spinal Injury Association Impairment Scale.

c UEMS: Upper Extremity Motor Score.

d ISNCSCI: International Standards for Neurological Classification of Spinal Cord Injury.

e LEMS: Lower Extremity Motor Score.

f ECT: Trunk Control Scale.

g INR LGII: Instituto Nacional de Rehabilitación Luis Guillermo Ibarra Ibarra.

Researchers include rehabilitation type as a predictor variable since it is part of the context of the patient preceding and affecting outcomes. Due to the observational nature of the study, researchers do not assume modifications in rehabilitation treatment between model application and outcome assessment. Researchers will conduct sensitivity analyses to evaluate potential effect modification on SCIM-III prediction by stratification by rehabilitation type.

### Data Collection Procedure

During the first phase of the study, trained personnel started in October 2025, retrospective data collection from the INR LGII institutional electronic clinical records system covering the period from January 2015 to June 2026, following a standardized data extraction protocol. Retrospective data will be used to train ML models. Three board-certified rehabilitation physiatrists with ≥2 years of experience in SCI rehabilitation are responsible for predictor assessments (AIS/ISNCSCI classification and ECT scoring) at INR LGII. Prior to data collection, evaluators will complete a standardized calibration session to ensure interrater consistency, documented in the data quality manual. All records will be anonymized at extraction, replacing personal identifiers with study codes. Investigators will define in the data quality manual procedure for data entry, missing data handling, range checks, and interrater consistency for clinically judged variables. As mentioned before, an external validation cohort of 100 new patient records will be prospectively recruited (October 2026-October 2027), consistent with recommendations for temporal validation in clinical ML studies [25,26]. During this prospective component (2026‐2027), new participants admitted to INR LGII who meet the eligibility criteria will be prospectively enrolled in the external validation cohort. Participants will be initially evaluated to determine all clinical variables (AIS, UEMS, LEMS, and ECT) at 3, 6, and 12 months post admission.

In those individuals, clinical investigators will evaluate SCIM-III following the Catz et al [16] protocol. Two board-certified rehabilitation physiatrists with experience in SCI rehabilitation will administer SCIM-III assessments. Evaluators will be blinded to predictor values at the time of SCIM-III evaluation. Admission variables (AIS, UEMS, LEMS, and ECT) will not be accessed during outcome measurement, preventing label leakage. Interrater reliability will be established through a standardized calibration session. Blinding procedures will be documented in the data quality manual. Clinical records used in the prospective component will not overlap with datasets used previously for the development of the models. No patient record from the prospective cohort will appear in the retrospective development dataset (2015‐2026), ensuring complete independence between development and validation data. Investigators will analyze any dataset differences to assess model generalizability.

Investigators will store data in password-protected institutional equipment, complying with Mexico’s Ley General de Protección de Datos Personales en Posesión de Sujetos Obligados and INR LGII ethical requirements.

### ML Model Development (Preprocessing)

Prior to model development, the dataset will undergo descriptive statistical analysis to examine the distributions, frequencies, and patterns of missing data. Exploratory correlation analyses will be conducted using Pearson and Spearman coefficients and correlation matrices to identify relevant predictors of SCIM-III and detect multicollinearity among variables.

Handling of missing data: a 2-step imputation strategy will be applied to address the missing data. Variables with less than 10% missing data will be addressed using median or mode imputation according to their distribution; those with 10%‐30% missingness will receive multivariate regression imputation with predictive mean matching. Records with more than 30% missing data in the defined predictor variables will be excluded according to the elimination criteria. Sensitivity analyses will compare the imputed datasets with complete case analyses to assess the stability of imputation decisions.

Feature preprocessing and encoding: categorical variables will be encoded as follows: AIS grades via one-hot encoding to avoid artificial ordinality, sex as a binary variable (0/1), and type of rehabilitation as a nominal variable with one-hot encoding. Continuous variables, including UEMS, LEMS, ECT, age, and time since injury, will undergo range validation, followed by *z* score normalization for the multilayer perceptron (MLP), minimum-maximum scaling for tree-based models, and Gaussian process regression (GPR). If the correlation analysis detects predictor redundancy, dimensionality reduction using principal component analysis (PCA) will be applied. All preprocessing decisions will be recorded in a version-controlled log to ensure complete reproducibility of the results. All preprocessing parameters (imputation values, normalization coefficients, and encoding mappings) will be derived from the training data and stored as part of the final model object for application to new observations without recalculation.

Variable selection: 3 complementary strategies will be combined: (1) filter methods using univariate statistics (Pearson/Spearman correlation and ANOVA *F* tests), (2) embedded methods based on model-derived importance (Gini impurity for trees, regularized coefficients for linear models), and (3) wrapper methods using recursive feature elimination with cross-validated performance as the selection criterion. The final inclusion of a predictor will require the convergence of at least 2 strategies and justification of clinical interpretability.

Researchers will apply all preprocessing steps uniformly across groups since no group-specific transformations are expected.

### Model Training

Six predictive model architectures will be trained and compared, ranging from interpretable statistical baselines to high-capacity nonlinear algorithms, with the goal of identifying the most suitable approach for predicting SCIM-III scores at 3, 6, and 12 months post admission. Given that SCIM-III is modeled as a continuous outcome in the primary analysis, model performance will be primarily evaluated using regression metrics, including root-mean-square error (RMSE), mean absolute error, and calibration measures (eg, prediction calibration and calibration plots), as well as prediction error quantified through prediction or CIs around the predicted scores.

Model architectures are as follows: (1) multivariate linear regression will serve as an interpretable statistical baseline; (2) decision trees (classification and regression tree [CART]) will generate transparent IF-THEN clinical rules with pruning and maximum depth constraints to limit overfitting; (3) categorical boosting (CatBoost; gradient boosting algorithm) natively handles categorical variables and class imbalances without the need for additional preprocessing; (4) light gradient boosting machine (LightGBM) will be tuned for the efficient handling of large data volumes and predictor importance ranking; (5) an MLP will be implemented with 2 hidden layers, Rectified Linear Unit or Leaky Rectified Linear Unit activation functions, Adam or Stochastic gradient descent optimization, and batch normalization; and (6) GPR will provide probabilistic predictions with 95% calibrated credible intervals to quantify predictive uncertainty.

Hyperparameter optimization: an initial random search will explore the hyperparameter space, and Bayesian optimization will perform fine-tuning. The architecture and regularization parameters of the MLP, depth and splitting criteria of CART, learning rates and regularization terms for CatBoost and LightGBM, and kernel selection for GPR will be adjusted based on performance in 10-fold stratified cross-validation.

Implementation and reproducibility: all models will be implemented in Python using scikit-learn, TensorFlow/Keras, or equivalent libraries with fixed random seeds. The dataset will be split according to a 60/20/20 (training/validation/test) scheme, with 10-fold stratified cross-validation applied during training. Investigators will allocate all records from the same individual (initial, 3, 6, and 12 months) to the same partition (training, validation, or test) and to the same fold during 10-fold cross-validation to prevent participant-level data leakage across subsets. Codes, model configurations, and preprocessing pipelines will be made available on Open Science Framework (OSF) and GitHub after publication in accordance with JMIR’s open science requirements.

### Overfitting Prevention

To ensure generalizable models and mitigate overfitting—where models learn noise rather than underlying relationships—a multifaceted strategy will be implemented throughout development, structured around 6 approaches.

#### Data-Centric Regularization

The following data-centric strategies will be implemented to reduce overfitting and improve model generalizability:

- Hold out: a rigorous data partitioning strategy will be used from the outset. The dataset will be divided into training (60%), validation (20%), and test (20%) sets. The validation set will be used exclusively for hyperparameter tuning and model selection, while the test set will be held back entirely until the final model evaluation to provide an unbiased estimate of its real-world performance.

- Cross-validation: all model training and hyperparameter optimization will be guided by stratified 10-fold cross-validation on the training set. This technique partitions the training data into 10 complementary subsets, iteratively training on 9 and validating on 1. This maximizes the use of available data for training and provides a more robust and less noisy estimate of model performance than a single train/validation split, making the model selection process more resilient to overfitting.

#### Algorithmic Regularization Techniques

Model-specific regularization will be a cornerstone of our approach to constrain model complexity:

- For neural networks (MLP): we will use dropout (randomly ignoring a fraction of neurons during training), L1/L2 weight regularization (penalizing large weights), batch normalization (stabilizing layer inputs), and early stopping (halting training when performance on the validation set ceases to improve).
- For decision trees (CART): tree growth will be constrained using prepruning (eg, limiting maximum depth or minimum samples per leaf) and post pruning (cost-complexity pruning) to prevent the tree from becoming overly specific to the training data.
- For ensemble methods (CatBoost and LightGBM): we will use shrinkage (learning rate) to slow down the learning process and L1/L2 regularization terms directly within the boosting objective function to penalize model complexity.
- For GPR: the flexibility of the model will be controlled by selecting appropriate kernels and incorporating a noise kernel to explicitly model and account for irreducible noise in the data, preventing the model from fitting to it.

#### Feature Selection and Dimensionality Reduction

As detailed in the preprocessing section, rigorous feature selection using filter, wrapper, and embedded methods will eliminate redundant predictors, reducing the model’s “degrees of freedom” and lowering overfitting risk. If predictor redundancy is high, PCA will be considered to compress the feature space while retaining the signal.

To ensure that no information from validation or test data contaminates model development, all preprocessing steps—including missing data imputation, normalization/scaling, feature encoding, dimensionality reduction (PCA), and feature selection—will be performed exclusively within the training partition. During k-fold cross-validation, these transformations will be fitted on each training fold and applied to the corresponding held-out fold without refitting, ensuring that no statistics computed from validation data influence the model. The same fitted preprocessing parameters from the final training set will be stored and applied unchanged to the external validation cohort. Hyperparameter tuning will likewise rely solely on cross-validated performance within the training data. This workflow ensures that performance estimates reflect true generalization rather than optimistic bias from information leakage.

#### Simplicity via the Principle of Parsimony

Model selection will not be based solely on raw predictive performance (eg, *R*^2^). We will explicitly consider model complexity using information criteria (for linear models) and by comparing the performance of simpler, more interpretable models (eg, multivariate linear regression and shallow decision trees) against more complex ones. A more complex model will only be selected if it provides a substantial and clinically meaningful improvement in performance on the validation set, thereby favoring simpler and inherently more generalizable solutions when their performance is comparable.

#### Conservative Hyperparameter Optimization

The hyperparameter search space will be carefully defined to avoid extreme configurations known to cause overfitting (eg, excessively deep trees and very high learning rates). Optimization, guided by cross-validation performance, will explicitly minimize validation error, not training error. Bayesian optimization is particularly advantageous here, efficiently exploring the trade-off between complexity and performance.

#### Final and Unbiased Performance Evaluation

Upon completion of all development and tuning using the retrospective cohort, the final optimal models will be evaluated on the prospective external validation cohort (n=100, recruited October 2026-October 2027). This temporal separation provides an unbiased estimate of true generalizability and expected clinical performance, the ultimate safeguard against inadvertent overfitting during iterative development. Researchers will examine model stability through bootstrap resampling (1000 iterations) to quantify the variability of individual predictions and performance estimates across bootstrap samples.

By integrating these 6 strategies, the project will systematically minimize overfitting risk, ensuring the developed ML-based rules are robust and clinically useful for predicting functional independence after SCI.

### Model Evaluation

Model interpretability will be assessed using Shapley additive explanations (SHAP) values to quantify predictor contributions at both global and individual prediction levels. Investigators will analyze subgroup fairness by reporting model performance (RMSE, *R*²) stratified by sex, age group (18‐40, 41‐60, and >60 years), AIS grade, and time since injury. Also, researchers will report subgroup sample sizes and CIs, and if the RMSE ratio exceeds 1.5 across groups, investigators will explore recalibration within underperforming subgroups. Full model performance measures are included in Table 2.

**Table 2.** Performance metrics and clinical relevance for the evaluation of the predictive model.

Metric	Application	Clinical relevance
RMSE^a^	Regression accuracy	Prediction error in SCIM-III^b^ points
MAE^c^	Average absolute error	Interpretable error magnitude
*R*²	Variance explained	Model fit quality
AUROC^d^	Binary classification (functional threshold)	Discrimination between functional categories
Sensitivity/specificity	Threshold-based classification	Clinical decision support performance
Calibration plot+Hosmer-Lemeshow	Agreement between predicted and observed SCIM-III (continuous calibration); goodness-of-fit for threshold classifications	Model reliability for clinical decision-making
Decision curve analysis	Net benefit of using the model versus treat-all or treat-none strategies across a range of clinical decision thresholds	Clinical utility: justifies model adoption over simpler clinical rules.

a RMSE: root-mean-square error.

b SCIM-III: Spinal Cord Independence Measure, version III.

c MAE: mean absolute error.

d AUROC: area under the receiver operating characteristic curve.

Additional considerations for model evaluation are described below:

- A trivial statistical baseline (predicting the mean SCIM-III score) will define the minimum acceptable performance for the continuous prediction task. Models must significantly outperform this baseline using regression-based performance metrics (eg, RMSE, mean absolute error, *R*², and calibration) to demonstrate clinical usefulness.
- Secondary classification analyses will binarize SCIM-III scores using clinically meaningful thresholds. A score of ≥55 will represent functional independence sufficient for discharge planning, while ≥75 will indicate high independence. For these secondary dichotomized outcomes, performance will be summarized using the area under the receiver operating characteristic curve, sensitivity, specificity, and decision-curve analysis, and area under the curve (AUC) values will be compared using the DeLong test with 95% CIs.
- Both thresholds will be reported with clinical justification. The primary model output remains a continuous SCIM-III prediction, with classification analyses considered exploratory secondary outcomes.

### External Validation

The best-performing model will undergo external validation in a prospective independent cohort of 100 new patient records not used during training, recruited between October 2026 and October 2027. This temporal separation constitutes a more stringent test than random partitioning, as it captures potential shifts in patient characteristics or clinical practices over time [27]. The prospective validation cohort (2026‐2027) and the retrospective development cohort (2015‐2026) will share identical eligibility criteria, predictor definitions, and SCIM-III outcome protocol. Investigators will assess potential differences in case-mix, rehabilitation practice patterns, or data completeness between periods through descriptive comparison prior to model evaluation. All preprocessing parameters derived from the training set—including imputation values, normalization coefficients, and encoding mappings—will be applied without recalculation to the validation cohort to prevent information leakage.

Validation will assess calibration (calibration plots, Hosmer-Lemeshow test, Bland-Altman limits of agreement, and Pearson correlation between predicted and observed SCIM-III scores) and discrimination (RMSE and AUC) using unseen data. CIs (95%) will be estimated via bootstrap resampling (2000 iterations). Performance degradation exceeding 0.05 in AUC between internal and external validation will trigger a prespecified covariate shift analysis.

Model performance will be benchmarked against published prediction rules using comparable predictors [14]. Subgroup analyses will examine predictive accuracy across AIS grade, age groups, and time since injury to identify heterogeneity and support clinically tailored application of the prediction model.

### Model Output and AI-Specific Considerations

Models will output a continuous predicted SCIM-III score (0‐100) at 3, 6, and 12 months post admission, framing the primary task as regression. Classification labels will only appear in secondary analyses using clinically meaningful SCIM-III thresholds. GPR will additionally provide 95% credible intervals to quantify prediction uncertainty [17]. To enhance clinical transparency, individual predictions will be accompanied by SHAP values identifying the contribution of each predictor to the estimated SCIM-III score [28].

The model is intended as a clinical decision support tool to inform rehabilitation goal-setting and discharge planning, not to replace clinical judgment. Rehabilitation clinicians will enter 8 admission variables (age, sex, time since injury, AIS grade, UEMS, LEMS, ECT, and rehabilitation type). The backend will automatically perform preprocessing, encoding, and prediction. If predictor values are unavailable at the time of prediction, the system will flag the affected variables and apply the imputation parameters derived from the training set. Results will present a clinically interpretable SCIM-III estimate accompanied by its uncertainty interval. The prototype will initially be tested by investigators; clinician usability testing is planned in later project stages.

Because the primary task is regression, class imbalance methods are not required. For secondary classification analyses using SCIM-III thresholds, imbalance will be evaluated; if ratios exceed 3:1, stratified sampling and precision-recall metrics will complement the area under the receiver operating characteristic curve evaluation.

Researchers planned the model to be used by trained rehabilitation clinicians capable of reliably performing ISNCSCI classification, SCIM-III, and ECT scoring. An onboarding guide embedded in the application interface will orient new users. Users without formal ISNCSCI, SCIM-III, and ECT training should complete structured training prior to clinical use.

### Ethical Considerations

The study was reviewed and approved by the Research Committee and Ethics in Research Committee of the INR LGII (protocol INRLGII 128/25, approved October 21, 2025).

Study follows Helsinki Declaration and Mexican regulations [29]; retrospective anonymized records exempt consent. For prospective data collection, written informed consent will be obtained from all participants or their legal representatives. Participants will be clearly informed about the study objectives, procedures, potential risks, and the use of their clinical and rehabilitation data for developing and validating an ML-based predictive model. This study is classified as minimal risk, as it involves secondary analysis of existing clinical data without direct intervention. Nonetheless, potential psychological or social implications related to predictive outcomes will be carefully considered, particularly regarding how prognostic information may influence patient expectations. Predictive results will not be used as the sole basis for clinical decision-making. Personal identifiers will be removed, and datasets will be anonymized or deidentified prior to analysis. Data will be stored securely with restricted access, and safeguards will be implemented to prevent unauthorized use or re-identification. Data will be used exclusively for the purposes outlined in this protocol. Any secondary use will require additional ethical approval.

Formal patient and public involvement in study design was not incorporated, given the retrospective nature of the primary data source and the early development stage. Patient perspectives were informally considered during protocol design through consultation with INR LGII rehabilitation physiatrists who provided clinical input on SCIM-III threshold values and workflow integration. In the last year, a structured usability study with rehabilitation clinicians and patient advisory input will be conducted and reported separately. The authors acknowledge the significance of patient co-design in AI clinical tools and commit to incorporating patient and caregiver views before clinical deployment.

This study is preregistered on the OSF (DOI: 10.17605/OSF.IO/4FMY6) and on the Mexican national clinical registry [30]. Both registrations are completed prior to prospective data collection.

### Timeline and Work Plan

Project timeline, methodological stages, and milestones for the development, validation, and implementation of the SCIM-III prediction model are shown in Table 3.

**Table 3.** Timeline, work plan, and key milestones across project stages.

Stage	Dates	Activities	Milestone
1	October 2025-June 2026	Data collection, anonymization, database structuring, descriptive and exploratory statistical analysis, and first technical report	Structured database
2	June 2026-March 2027	Development and training of ML^a^ models (regression, DT^b^, ANN^c^, and GPR)^d^, k-fold cross-validation, model comparison, and congress presentation	Trained models+publication
3	June 2027-March 2028	External validation, digital application development, patent/IP registration, international congress (ASIA^e^ 2027), and training workshop	Validated rule+app prototype

a ML: machine learning.

b DT: decision tree.

c ANN: artificial neural network.

d GPR: Gaussian process regression.

e ASIA: American Spinal Injury Association.

### Team

The multidisciplinary team comprises 10 researchers across 3 institutions (Table 4).

**Table 4.** Research team members, institutional affiliations, and project roles.

Name	Institution/specialty	Role
Alberto Isaac Pérez-Sanpablo	INR LGII^a^/biomedical engineering	Principal investigator
Jimena Quinzaños-Fresnedo	INR LGII/neurological rehabilitation	Coinvestigator
Aída Barrera-Ortiz	INR LGII/neurological rehabilitation	Coinvestigator
Marlene A Rodríguez-Barragán	INR LGII/neurological rehabilitation	Coinvestigator
Fabiola Monserrat Palomino-Ramos	INR LGII/neurological rehabilitation	Coinvestigator
Alicia Meneses-Peñaloza	INR LGII/pediatric rehabilitation	Coinvestigator
Oscar Prado-Escobar	INR LGII/rehabilitation medicine (resident)	Student Investigator
Marcela D Rodríguez-Urrea	UABC^b^/computer science	Coinvestigator (ML^c^)
Irvin Hussein López-Nava	CICESE^d^/artificial intelligence	Coinvestigator (AI^e^)
José Ambrosio-Bastián	Independent/artificial intelligence	Coinvestigator (AI)

a INR LGII: Instituto Nacional de Rehabilitación Luis Guillermo Ibarra Ibarra.

b UABC: Universidad Autónoma de Baja California.

c ML: machine learning.

d CICESE: Centro de Investigación Científica y de Educación Superior de Ensenada.

e AI: artificial intelligence.

## Results

The study was funded by the Secretaría de Ciencia, Humanidades, Tecnología e Innovación grant CBF-2025-I-3891 on October 29, 2025. The study was approved by the institutional research and ethics committees on October 21, 2025 (INRLGII 128/25). Researchers began retrospective data extraction from the institutional electronic clinical records system (records from January 2015 to June 2026) in October 2025. As of the submission of this manuscript in March 2026, researchers extracted and anonymized 119 retrospective patient records meeting eligibility criteria. Researchers are performing quality control and preprocessing of this data. Researchers plan to begin ML model development in June 2026 and expect to complete it by March 2027. Researchers plan to open enrollment of the external validation cohort (n=100) in October 2026 and close it in October 2027. External validation is planned between June 2027 and March 2028. Results of the development and validation study are expected to be submitted for publication in January 2028.

## Discussion

### Principal Findings (Anticipated)

This protocol describes the development of the first ML-based predictive model for SCIM-III outcomes in a Mexican SCI cohort. We anticipate that including the ECT trunk control variable—not captured by the standard ISNCSCI—together with classic clinical variables (UEMS, LEMS, AIS, age, and time since injury) will meaningfully improve prediction accuracy over models based solely on neurological classification. Preliminary data from our group suggest that trunk control comprises a significant proportion of functional independence variance in this population [9].

Comparative models, interpreted through SHAP analysis, will identify the optimal balance between predictive accuracy and clinical interpretability for trustworthy rehabilitation decision support.

### Comparison With Previous Work

Numerous studies have been conducted on predicting recovery in individuals with SCI [31]. van Middendorp et al [7] developed a clinical prediction rule based on age and clinical neurological parameters, such as motor and sensory assessments, to predict the long-term likelihood of independent walking, and Kaminski et al [32] reported in 2017 that the 1-year functional outcome, as measured by the SCIM-III, can be estimated using a simple equation that considers four parameters from the initial physical examination. Similarly, predictive models based on ML have been developed to estimate the level of functionality in individuals from the acute stage, some using the Functional Independence Measure or the SCIM-III, concluding that it is possible to predict with good accuracy the gait and/or independence in activities of daily living [33,34]. Zhong et al [35] conducted a systematic review and meta-analysis to evaluate the performance and quality of ML models in predicting SCI prognosis. The research indicated that ML models are significantly superior to traditional statistical models. This cutting-edge approach provides insights into how early prediction can be achieved using clinical characteristics at the time of patient admission.

### Limitations

As this is a retrospective design, we may encounter unavoidable biases and lack control over confounding variables. The sample size is small for ML, and the study was conducted at a single center, resulting in a good model but not universally reliable. Although researchers planned a two-stage imputation strategy and sensitivity analyses, we cannot fully exclude the possibility of residual bias from missing data. Also, the relatively small development sample may limit stability for high-capacity architectures (MLP and GPR) despite the proposed overfitting prevention strategies, and performance may degrade in populations with different case-mix profiles.

Also, participation (International Classification of Functioning, Disability, and Health) was not measured in this study. As of March 2026, no ML model has been effectively used to predict the prognosis of individuals with SCI in clinical practice. Greater interpretability of a model allows clinicians to better understand its predictive value and make clinical decisions that benefit individuals. Another limitation of our study is that it does not include biomarkers or advanced neuroimaging; however, in low-income countries, most centers lack the technology to perform these procedures in the acute phase. It is hoped that future research will continue to address this complex problem of model interpretability.

### Conclusions

Early prediction of functional independence recovery after SCI allows for the development of a rehabilitation strategy in patient management from the acute stage. Using ML, we aim to develop a tool that will enable us to identify key clinical predictors and provide a decision support system, thus predicting the degree of independence regained upon admission to an acute rehabilitation center. The protocol develops the first functional prediction tool for SCIs based on ML in Mexico, allowing for personalized rehabilitation planning and preparation for clinical application, laying the foundation for future research in SCI rehabilitation.

## Supplementary material

10.2196/95236 Peer Review Report 1 Peer review report by Secretaría de Ciencia, Humanidades, Tecnología e Innovación (SECIHTI, Mexico)

## Abbreviations

AIS: American Spinal Injury Association Impairment Scale
AUC: area under the curve
CART: classification and regression tree
CatBoost: categorical boosting
ECT: Trunk Control Scale (Escala de Control de Tronco)
GPR: Gaussian process regression
INR LGII: Instituto Nacional de Rehabilitación Luis Guillermo Ibarra Ibarra
ISNCSCI: International Standards for Neurological Classification of Spinal Cord Injury
LEMS: Lower Extremity Motor Score
LightGBM: light gradient boosting machine
ML: machine learning
MLP: multilayer perceptron
MRI: magnetic resonance imaging
OSF: Open Science Framework
PCA: principal component analysis
RMSE: root-mean-square error
SCI: spinal cord injury
SCIM-III: Spinal Cord Independence Measure, version III
SHAP: Shapley additive explanations
UEMS: Upper Extremity Motor Score
