# Supplementary material for: Machine Learning–Based Predictive Model for Functional Independence in Spinal Cord Injury: Protocol for a Predictive Rule Development and Validation Study
Source: JMIR Res Protoc. 2026 Jul 3;15:e95236. doi: 10.2196/95236 (PMC13331064; doi:10.2196/95236)
Supplement: Peer Review Report 1 [file resprot-v15-e95236-s001.pdf]

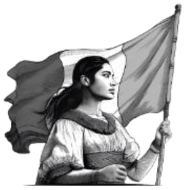

INRLGII/CI/392/2025
México a 3 de octubre de 2025
Asunto: Entrega de dictamen 01 CI

DR. ALBERTO ISAAC PÉREZ SANPABLO
INVESTIGADOR PRINCIPAL
PRESENTE

En respuesta a la solicitud de la revisión del protocolo**: Modelo predictivo de independencia funcional en lesión medular utilizando variables clínicas y aprendizaje automático,** le comunico que su proyecto fue sesionado el 29 de septiembre por el comité de ética en investigación y el 30 del mismo mes por este comité, quien emite el siguiente

DICTAMEN: REQUIERE MODIFICACIONES

Cabe señalar que usted cuenta con 2 semanas para realizar las modificaciones sugeridas, por lo que hacemos de su conocimiento las siguientes recomendaciones:

Respecto al dictamen del comité de investigación:

- *Favor de responder con una carta dirigida al comité de investigación anexando la respuesta a cada revisor de manera puntual, señalando cuál es la modificación que se hizo y en qué parte del protocolo se encuentra.*
- *El investigador puede no estar de acuerdo con algún comentario y tiene el derecho de no responder siempre y cuando lo justifique; esto, no tendrá repercusión alguna en el proceso de revisión.*
- *En el archivo electrónico señalar con comentarios o con amarillo el lugar de la modificación.*
- *Para cualquier duda o aclaración puede dirigirse a la mesa de control.*

Respecto al dictamen del comité de ética en investigación:

Por normatividad, el CEI, debe reportar los protocolos que fueron revisados, motivo por el cual es indispensable que rellene el formulario que se le enviará correo electrónico: https://forms.gle/CVFgqhQEJ34toveR6 para poder continuar la revisión por este Comité.

- *Responda de manera puntual a las observaciones, a cada inciso y señalando el párrafo y/o número de página en el que se hizo la modificación. Se adjunta la guía para la elaboración del consentimiento informado para su cotejo.*
- *En el archivo electrónico señalar con comentarios o con amarillo el lugar de la modificación.*

Sin otro particular, aprovecho la ocasión para enviarle un cordial saludo.

ATENTAMENTE

DRA. JIMENA QUINZAÑOS FRESNEDO
SECRETARIA TÉCNICA DEL COMITÉ

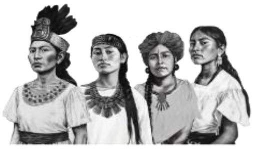

Dictamen protocolo: Modelo predictivo de independencia funcional en lesión medular utilizando variables clínicas y aprendizaje automático

Comentarios menores: referencias, ortografía, redacción.
Comentarios mayores: metodológicos

REVISOR 1

1. El proyecto plantea una clara pregunta de investigación coherente con la hipótesis propuesta. El marco teórico y antecedentes dejan claramente establecido el estado del arte para enmarcar el problema de investigación.
2. El diseño propuesto observacional, analítico, mixto (retrospectivo-prospectivo), y metodología propuesta es adecuado para abordar la pregunta establecida.
3. De acuerdo a las variables establecidas el análisis estadístico se ajusta al objetivo.
4. El proyecto puede aportar conocimiento relevante para atender una problemática actual.

REVISOR 2

- Dentro de la hipótesis se comenta que se buscará un modelo de predicción que sea confiable, especificar cómo se evaluará la confiabilidad del modelo de predicción. Sugiero se considere dentro de la hipótesis algún valor de desempeño predictivo esperado para el modelo.
- En antecedentes sugiero profundizar más sobre los rendimientos que tienen los algoritmos reportados en la literatura para entender mejor la necesidad de desarrollar nuevos modelos predictivos.
- Especificar si se correrá un modelo predictivo para cada horizonte de tiempo o si se hará un análisis conjunto de todos los puntos de tiempo.
- Detallar cómo es que se visualiza que este algoritmo se aplicará en la práctica clínica y de qué forma contribuirá a mejorar la atención de los pacientes con lesión medular.

Este es un ejemplo para dar respuesta; usted puede decidir el mejor formato para hacerlo, incluso puede responder enseguida de cada observación del revisor, siempre y cuando incluya los apartados a responder: comentario, respuesta y ubicación de la modificación en el documento.

REVISOR	COMENTARIO DEL REVISOR	RESPUESTA DEL INVESTIGADOR	UBICACIÓN EN EL DOCUMENTO

Ciudad de México a 03 de octubre de 2025

DICTAMEN DEL COMITÉ DE ÉTICA EN INVESTIGACIÓN

Documentos revisados:

1) Propuesta de Protocolo de Investigación. Modelo predictivo de independencia funcional en lesión medular utilizando variables clínicas y aprendizaje automático
2) Consentimiento Informado, versión 01.

Investigador principal:	**DR. ALBERTO ISAAC PÉREZ SANPABLO**
La investigación se llevará a cabo en:	Instituto Nacional de Rehabilitación Luis Guillermo Ibarra Ibarra.
**Acuerdo:**	**CEI-09-IX-ORD-2025**
**DICTAMEN:**	**Pendiente de aprobación.**<br>Investigación con riesgo mínimo.<br>Art. 17 fracción III, RLGSMIS

Comentarios generales:

a. Favor de incluir en la carta respuesta que proporcione a este comité, una aclaración sobre lo siguiente:

   ¿Cuál será el procedimiento o medio por el que el grupo de investigadores obtendrán el consentimiento informado de cada participante? Especificar, quién lo hará, cómo lo hará, etc.

b. **LENGUAJE TÉCNICO.** *El consentimiento está redactado de una manera muy técnica, es decir, utiliza un lenguaje complicado de entender y como se menciona en el instructivo para la elaboración del consentimiento informado:*

> *De acuerdo con la Guía nacional para la integración y funcionamiento de los Comités de Ética en Investigación que emite la Comisión Nacional de Bioética, el lenguaje que se emplee a lo largo del consentimiento informado deberá ser entendido por un estudiante con nivel de educación primaria.*

Motivo por el cual solicitamos que reformule el consentimiento tomando en cuenta las siguientes consideraciones:

c. Se identificó que hay párrafos donde se menciona derrame cerebral y no lesión medular. Favor de corregir.

d. Favor de confirmar esta información: se trata de un estudio retrospectivo, por lo que según el protocolo sólo se invitará a los pacientes que les falten evaluaciones para completar los periodos mencionados (3, 6 y 12 meses).

e. Favor de hablar con el término "investigación" cuando se refiere al proyecto al que se le está invitando al sujeto a participar, evitando así la confusión con algunos procedimientos o estudios que forman parte de ella (análisis, tomografías, etc.)

Respecto al consentimiento informado se hacen las siguientes sugerencias en relación a cada apartado del formato institucional.

3. ¿Por qué es importante esta investigación? Señala de manera clara y sencilla la justificación de la investigación.

- *Menciona derrame cerebral y no lesión medular, favor de aclarar o corregir.*

4. ¿Por qué he sido invitado a participar en esta investigación? Enlista breve y claramente los criterios de inclusión al protocolo.

- El lenguaje de este apartado es muy técnico; favor de replantear. Se sugiere enlistar los criterios:
- Favor de eliminar la oración "*Y puede entender y firmar este consentimiento para participar de forma voluntaria*" ya que si no puede entenderlo ustedes no lo invitarían.

Usted escribió:

> *"Usted fue invitado a participar en esta investigación porque tiene más de 18 años, ha tenido una lesión medular (LM) y su información está registrada en el Instituto Nacional de Rehabilitación. Además, ya cuenta con al menos una evaluación funcional llamada SCIM III y su expediente tiene los datos necesarios para ayudar en este estudio. También puede participar en una nueva evaluación funcional SCIM III que nos permitirá entender mejor su proceso de recuperación. Y puede entender y firmar este consentimiento para participar de forma voluntaria."*

Recordando que usted es el experto, se le sugiere tomar en cuenta lo siguiente:

Usted fue invitada o invitado a participar porque cumple con las siguientes características:

- Pacientes de este instituto, mayor de 18 años.
- Diagnóstico confirmado de lesión medular en fase subaguda o crónica (se lo confirma su médico)
- Ha tenido un seguimiento completo con evaluaciones a 3, 6 y/o 12 meses.
- Según su expediente, podemos obtener información sociodemográfica y funcional, es decir, por un lado, podemos conocer su sexo, etnia, nivel educativo, la ocupación, etc. y por otro lado las características de su enfermedad, es decir, qué tanto puede moverse, qué tipo de rehabilitación ha tenido, cómo ha evolucionado, su nivel de independencia o qué tan grave es su enfermedad (esto se sabe por las pruebas que le han realizado como: SCIM-III, AIS, UEMS, LEMS, ECT)

6. ¿En qué consistirá mi participación y cuánto durará?

Usted escribió:

"Su participación consistirá en permitir de manera anónima el uso de los datos clínicos y funcionales disponibles en su expediente electrónico, y en una evaluación que consistirá en la aplicación de la escala SCIM III, la cual será realizada por personal médico. La evaluación dura entre 10 y 15 minutos, no implica riesgos físicos ni invasivos, y se aplicará en una sola sesión."

- La información es precisa; sin embargo, el lenguaje es técnico.
- Favor de explicar en términos sencillos a qué se refieren con la escala SCIMM III
- Se sugiere sólo generalizar el uso de datos de su expediente digital
- En este apartado no es necesario mencionar los riesgos

7. ¿Cuáles son los beneficios de formar parte de esta investigación? Explicar brevemente si hay beneficios; si no los hay mencionarlo y señalar los beneficios indirectos.

- Debido a que la investigación busca beneficiar en un futuro; es importante ser claros y mencionar que no habrá beneficios para él o la participante; y después sí mencionar lo que en un futuro se espera.

Recordando que usted es el experto, se le sugiere tomar en cuenta lo siguiente:
Usted no obtendrá ningún beneficio por participar en esta investigación; sin embargo, en un futuro esperamos que con los resultados que obtengamos, logremos contribuir a generar conocimiento útil para mejorar la atención de futuros pacientes con lesión medular como usted.

8. ¿Existe alguna alternativa que pueda proporcionarme mayor beneficio de lo que me propone esta investigación?
Usted escribe:

> *No. Esta investigación no reemplaza su tratamiento, solo busca analizar información que ya existe o que se obtiene en una evaluación sencilla. No hay otra alternativa que le ofrezca mayor beneficio que participar voluntariamente para apoyar el desarrollo de una herramienta que podría ayudar a mejorar la atención de personas con LM.*

- Está clara la primera parte del aprtado.
- La segunda oración se percibe coercitiva; favor de eliminarla: “*No hay otra alternativa que le ofrezca mayor beneficio que participar voluntariamente para apoyar el desarrollo de una herramienta que podría ayudar a mejorar la atención de personas con LM*”

14. ¿En qué casos se me puede suspender de la investigación?

- Favor de eliminar las leyendas dirigidas al investigador*: (Explicar los criterios de eliminación)*

ATENTAMENTE

COMITÉ DE ÉTICA EN INVESTIGACIÓN
